# Design and Performance Study of a Compliant Mechanism Capable of Achieving Dual-Range Constant Force Output

**DOI:** 10.3390/mi17040417

**Published:** 2026-03-29

**Authors:** Chongchong Xu, Zengyun Liu, Yan Liu, Shuaishuai Lu, Zhiming Zhang, Fei Wang, Pengbo Liu, Peng Yan, Yingyue Yin

**Affiliations:** 1 Shandong Key Laboratory of CNC Machine Tool Functional Components, School of Mechanical Engineering, Qilu University of Technology (Shandong Academy of Sciences), Jinan 250353, China; 2 Shandong Institute of Mechanical Design and Research, Jinan 250031, China; 3 Department of Mechanical and Electrical Engineering, Heze Vocational College, Heze 274000, China; 4 Department of Intelligent Manufacturing, Shangdong Labor Vocational and Technical College, Jinan 250300, China; 5 School of Mechanical and Electrical Engineering, Jining University, Qufu 273155, China; 6 Key Laboratory of High-Efficiency and Clean Mechanical Manufacture, Ministry of Education, School of Mechanical Engineering, Shandong University, Jinan 250061, China

**Keywords:** compliant constant-force mechanism, dual-range constant-force output, Z-shaped beam, bistable beam

## Abstract

A compliant constant-force mechanism (CCFM), known for its frictionless, contact-free operation and inherently constant output, is typically limited to a single force range, restricting its adaptability to multi-task applications. To address this problem, in this study, we propose a dual-stage compliant constant-force mechanism (DSCCFM) that delivers a continuous dual-range constant-force output within a monolithic structure. The design integrates a Z-shaped beam with a bistable beam and a bistable rhombic beam, thereby forming the DSCCFM. By integrating the pseudo-rigid-body model (PRBM) with the chained-beam constraint model (CBCM), a theoretical model of the DSCCFM is established. Using a finite-element response surface model and multi-objective genetic algorithm (MOGA) optimization, the constant-force stroke was improved by approximately 38% over the initial design. The experiments confirm stable outputs of 6.72 N and 21.91 N across the 2–5.8 mm and 11.6–14.8 mm ranges, respectively. The DSCCFM effectively supports multi-stage force execution, cell gripping, and micro/nano-scale manipulation.

## 1. Introduction

In modern mechanical engineering and intelligent equipment systems, precise force control and smooth motion characteristics are becoming increasingly important [1,2,3]. The CCFM achieves force, motion, and energy transfer through the elastic deformation of flexible components [4,5,6,7]. Compared with traditional rigid constant-force mechanisms, it can stably output a constant force within a specific working stroke without the need for complex sensors and control systems, simplifying design and reducing costs. At the same time, it avoids the impact and wear caused by rigid contact and improves the stability and reliability of the system [8,9,10].

At present, CCFM design research mainly focuses on single-range constant-force output, and structural forms can be divided into parallel and series types. The parallel mechanism achieves a stable constant-force output by paralleling negative-stiffness and positive-stiffness modules to achieve zero stiffness within a specific stroke [11,12,13,14,15]. Qin et al. connected a bistable beam in parallel with a V-shaped beam and achieved a constant output force of approximately 45.5 N within a displacement range of 1.2 mm-2.2 mm [12]. Additionally, Tian et al. improved the bistable beam structure by designing a trapezoidal beam with holes and connecting it in parallel with a Z-shaped beam, achieving a stable constant force of 10 mN within a stroke of 0.5 mm [14]. This type of mechanism commonly uses a guiding design with inclined straight beams and straight beams sharing a fixed end, which improves motion accuracy. However, due to its short working stroke and complex structure, it is only suitable for short-distance clamping. In contrast, the series-type constant-force mechanism achieves constant output force by sequentially connecting negative-stiffness and positive-stiffness modules to transfer deformation step-by-step, utilizing the stiffness evolution characteristics [16,17,18]. Chen et al. proposed a nonlinear performance index based on the secondary buckling mode of flexible beams, providing a theoretical basis for the design of constant-force mechanisms [18]. Although the stroke of a series mechanism can be extended, its accuracy and stability are usually lower than those of a parallel structure. For scenarios such as precision assembly and biological tissue detection that require multi-level constant force, researchers have achieved constant-force output control by adjusting preload displacement [19,20] or a modular assembly strategy [21]. However, switching between different constant-force levels still requires assembling or disassembling modules, which limits efficiency and flexibility.

To improve performance, researchers have introduced intelligent design methods such as MOGA [22,23,24,25], topology optimization [26,27], and particle swarm optimization [28] to optimize structural stiffness, stress distribution, and motion performance, effectively improving the stability and range of the constant-force output [29,30,31,32,33,34]. However, existing institutions still face the problems of insufficient constant-force stability and limited travel, and the mainstream single-pole constant-force mechanisms have fixed intervals, making it difficult to adapt to cross-scale and multi-task operations, requiring frequent adjustment of execution units and increasing operational complexity. To solve this problem, in this study, we propose a hybrid configuration design based on the principle of a positive- and negative-stiffness combination and develop a DSCCFM through finite-element optimization. A wide-range segmented constant-force output and excellent stability are achieved, providing innovative technical support for adaptive constant-force applications such as cell clamping and robot operation.

The framework of this paper is as follows: in Section 2, the analysis and design of the mechanism principle are elaborated upon; in Section 3, the mechanical model of the constant-force mechanism is established; in Section 4, the parameter analysis and finite-element optimization simulation are discussed; in Section 5, the experimental verification of the mechanism is presented; and in Section 6, this study’s conclusions are summarized.

## 2. Mechanism Design

A traditional CCFM can usually only provide a single constant-force range, making it difficult to adapt to diverse task requirements and limiting their flexibility. To enhance the adaptability of the mechanism and reduce system complexity and production costs, designing a compliant mechanism with a dual-stage or multi-stage constant-force output has important application value.

**Figure 1:**
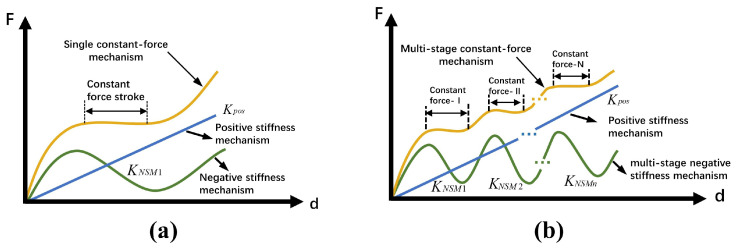
Principle of the constant-force mechanism: (**a**) single-stage constant-force mechanism; (**b**) multi-stage compliant constant-force mechanism.

As shown in Figure 1, the single-stage CCFM exhibits a zero-stiffness plateau on the force–displacement curve (dF/dx = 0, i.e., CCFM mode), where the output force remains constant and independent of displacement, thereby enabling a stable and non-destructive operation. A multi-stage constant-force mechanism extends this principle by establishing multiple independent constant-force intervals within a single motion range. Through the combination of compliant components with different stiffness characteristics and geometric constraints, nonlinear responses can be introduced to generate multiple zero-stiffness states in different displacement intervals, thereby producing multiple constant-force levels. Under this design principle, the number of constant-force regions can be increased by extending the staged stiffness-compensation process so that the stiffness-matching condition is satisfied successively over multiple target displacement intervals. As a result, the mechanism can generate corresponding two-stage, three-stage, or even higher-order constant-force outputs.

Based on the above working principle, the proposed mechanism is constructed using a positive–negative-stiffness combination approach and is composed of a positive-stiffness mechanism and negative-stiffness mechanisms with complementary characteristics at different deformation stages [35]. The compliant substructures are selected according to their respective mechanical roles in the overall mechanism. Specifically, the positive-stiffness mechanism is required to provide a stable and approximately linear restoring force over the working range, whereas the negative-stiffness mechanisms are required to exhibit sequential nonlinear responses capable of compensating for the positive stiffness in different displacement intervals. For the present dual-stage design, two negative-stiffness units are integrated and arranged to be activated successively during deformation, so that stiffness compensation can occur in two separate stages. Meanwhile, the positive-stiffness unit provides a linearly increasing reference force with displacement. As a result, the increasing force contributed by the positive-stiffness mechanism is dynamically offset by the staged decreasing forces generated by the two negative-stiffness units, thereby satisfying the following stiffness-matching relationship:

(1) $$K_{NSM1}=K_{NSM2}=-K_{pos}$$

**Figure 2:**
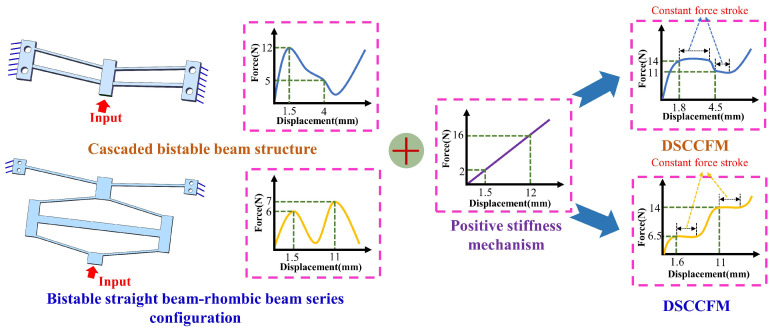
Force–displacement curves of two types of dual-negative-stiffness assemblies.

Bistable beams are widely employed as negative-stiffness units in mechanism design. To achieve dual-negative stiffness, two negative-stiffness units are integrated. However, the parallel bistable beam structure has notable limitations. Its maximum deformation is constrained with the overall stroke equivalent only to that of a single beam, and the adjustability of the second segment of its negative stiffness curve is limited, making it hard to adapt to large-stroke applications. To resolve this issue, this study adopts a configuration in which a bistable beam and a bistable rhombic beam are arranged end-to-end. This connection is adopted because the two units can be triggered successively during deformation, so that the first and second negative-stiffness responses can appear in different displacement intervals. This combined mechanism not only exhibits significantly superior deformation capacity compared with the parallel bistable structure but also features greater adjustability for the second segment of its negative-stiffness curve, thereby effectively meeting the demands of large-stroke applications (as shown in Figure 2).

**Figure 3:**
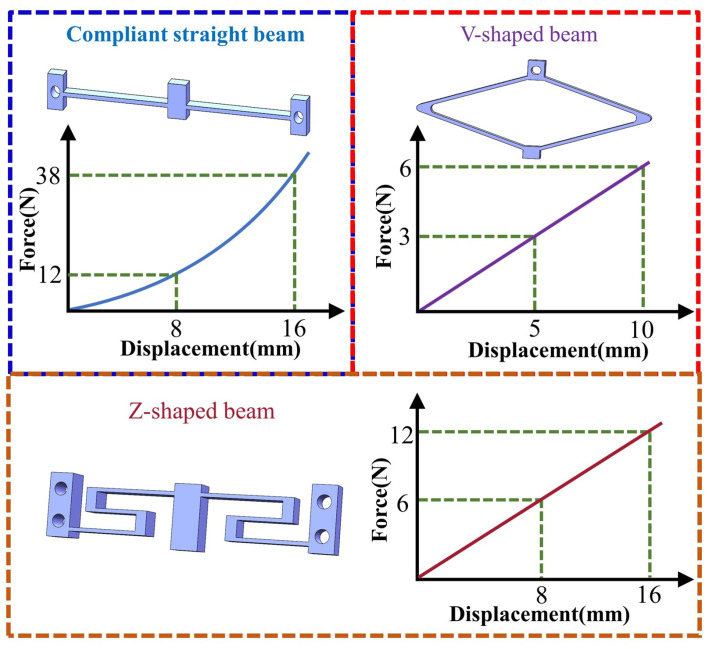
Force–displacement characteristics of different positive-stiffness mechanisms.

**Figure 4:**
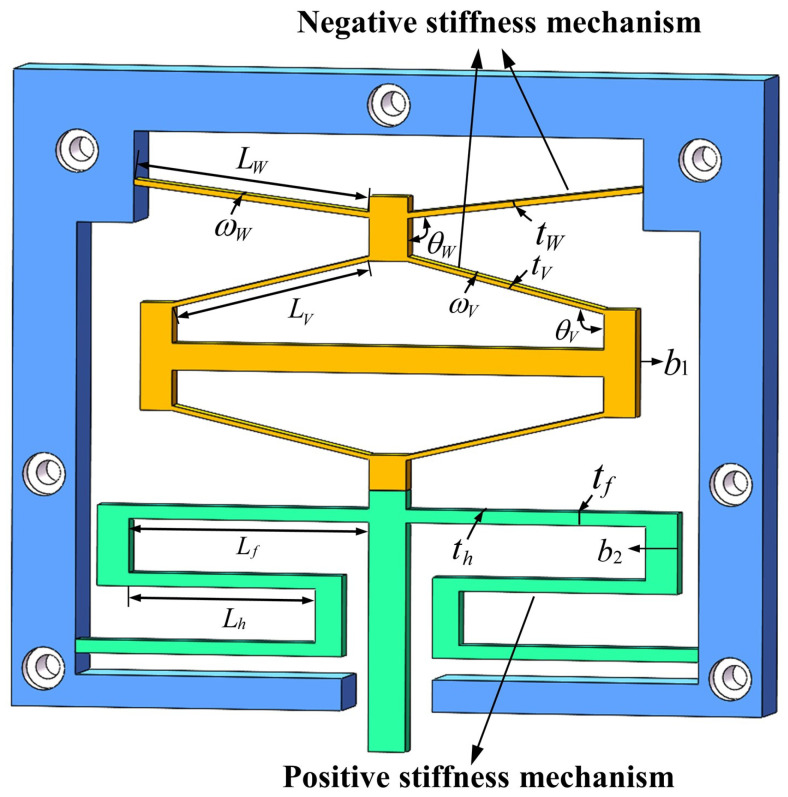
Dual-stage constant-force compliant mechanism.

As shown in Figure 3, compliant straight beams exhibit strongly nonlinear force–displacement characteristics under large deformation conditions. Under the constraint of equal volume, Z-shaped beams demonstrate better linear deformation capability than V-shaped beams, which is more conducive to ensuring the stability and consistency of the positive-stiffness output. Considering both the deformation adaptability of the mechanism and the requirement for linearity, a Z-shaped beam was adopted as the positive-stiffness unit, and a DSCCFM was constructed in parallel based on the aforementioned combination method of positive and negative stiffness, as shown in Figure 4. The mechanism adopts a parallel configuration, in which the Z-shaped beam and the combined bistable module achieve displacement compatibility and stiffness superposition, thereby reducing stiffness fluctuations. Its stiffness compensation exhibits distinct stage-dependent characteristics. Specifically, in the initial deformation stage, negative-stiffness compensation is mainly provided by the bistable beam, forming the first constant-force interval. When the deformation exceeds a critical threshold and the bistable beam enters the post-buckling stage, the bistable rhombic structure subsequently provides negative-stiffness compensation, thereby establishing the second constant-force stroke.

## 3. Modeling Analysis

The constant-force mechanism is designed based on the principle of stiffness compensation, incorporating a combination of positive and negative stiffness. Specifically, the positive stiffness is provided by a Z-shaped beam, while the dual-stage negative stiffness is realized by concatenating a bistable beam and a bistable rhombic beam in an end-to-end series configuration. In this section, individual theoretical models for the positive- and negative-stiffness modules are first developed.

### 3.1. Theoretical Model of the Positive-Stiffness Mechanism

**Figure 5:**
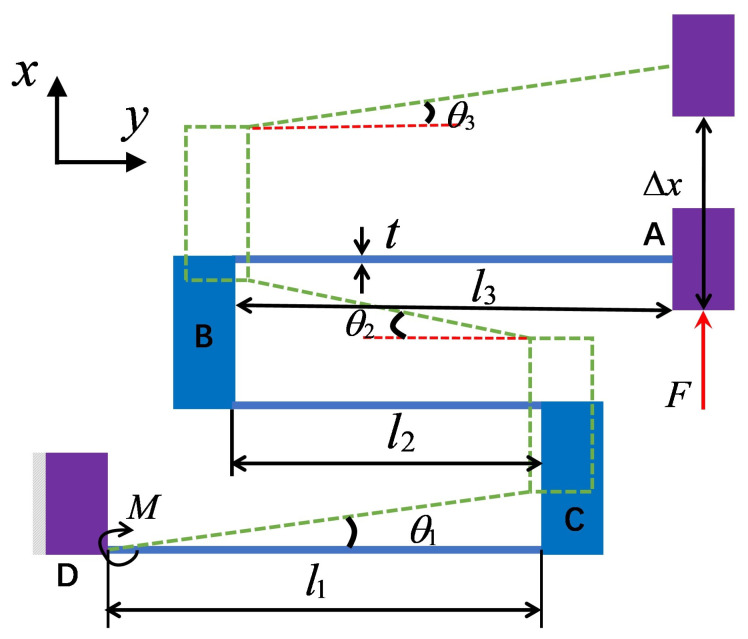
Schematic diagram of Z-beam deformation under force.

As shown in Figure 5, this constant-force mechanism provides positive-stiffness characteristics through a Z-shaped beam structure [36]. The structure consists of three flexible beams and two rigid blocks (B and C). Among the parameters, $l_{1}$, $l_{2}$, and $l_{3}$ represent the lengths of each flexible beam; *b* denotes the out-of-plane width of the Z-shaped beam; *F* stands for the thickness of the Z-shaped beam; and m is the torque at the fixed end D of the Z-shaped beam. When subjected to a force *F*, the guide block A produces an upward vertical displacement Δx, and at this point, the included angles generated after the deformation of the three flexible beams are $\theta_{1}$, $\theta_{2}$, and $\theta_{3}$, respectively.

For a cantilever beam, when a force *F* acts on the end of the beam, the following equations can be derived using mechanics of materials based on the boundary conditions:

(2) $$\frac{Fl^{2}}{2EI}-\frac{Ml}{EI}=0$$

(3) $$\frac{Fl_{3}}{3EI}-\frac{Ml^{2}}{2EI}=\delta x$$

Among them, δx is the output displacement, *M* represents the torque at the fixed end of the beam, *l* is the length of the flexible beam, *E* is Young’s modulus of the flexible beam material, and $I=bt^{3}/12$ is the moment of inertia of the flexible beam. Based on the above set of equations, the following can be obtained:

(4) $$\delta x=\frac{Fl^{3}}{12EI}$$

where $F_{\theta i}$ denotes the output force of each flexible beam in the Z-shaped beam. Based on the above equation, the following can be obtained:

(5) $$F_{\theta i}=\frac{7EI\theta_{i}}{{l_{i}}^{2}}$$

Based on the geometric relationships of the Z-shaped beam, the following equations can be derived:

(6) $$\left\{\begin{array}{c} l_{1}\left(1-\cos\theta_{1}\right)-l_{2}\left(1-\cos\theta_{2}\right)+l_{3}\left(1-\cos\theta_{3}\right)=0\\ l_{1}\sin\theta_{1}+l_{2}\sin\theta_{2}+l_{3}\sin\theta_{3}=\Delta x\\ \theta_{1}=\theta_{3} \end{array}\right.$$

Since $l_{1}$ and $l_{3}$ are of equal length, the positive stiffness of the Z-shaped beam is given by the following:

(7) $$K_{pos}=\frac{2Ebt^{3}}{2l_{1}^{3}+l_{2}^{3}}$$

It can be concluded from the above relationships that the length and width of the rigid block have no significant impact on the positive-stiffness characteristics of the Z-shaped beam. Therefore, when using finite-element methods to optimize the positive-stiffness mechanism, the above parameters can be ignored.

### 3.2. Theoretical Model of the Negative-Stiffness Mechanism

**Figure 6:**
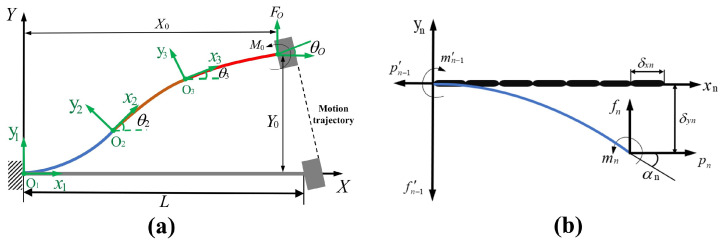
Modeling of the bistable beam: (**a**) overall analysis of the segmented bistable beam; (**b**) discretization of the bistable beam.

For the bistable beam and bistable rhombic beam, due to their symmetry, half of the bistable beam and a quarter of the composite trapezoidal beam are selected for analysis. The bending deformation of the compliant beam is nonlinear; thus, the CBCM is adopted to model and analyze the negative-stiffness mechanism [37]. Each compliant beam is divided into three equal beam elements connected end-to-end, and each beam element is modeled and analyzed. Its mechanical analysis is shown in Figure 6. The transverse force, the axial force, and the bending moment that act on the end of the compliant beam are denoted by $F_{O}$, $P_{O}$, and $M_{O}$, respectively; $X_{O}$, $Y_{O}$, and $\theta_{O}$ represent the end displacement and rotation angle of the compliant beam, and *L* denotes the length of the compliant beam along the *x*-axis. The beam constraint equations can be expressed as follows:

(8) $$\left(\begin{array}{c} f_{0}\\ m_{0} \end{array}\right)=\left[\left(\begin{array}{cc} 12 & -6\\ -6 & 4 \end{array}\right)+\frac{P_{0}L^{2}}{EI}\left(\begin{array}{cc} \frac{6}{5} & -\frac{1}{10}\\ -\frac{1}{10} & \frac{2}{15} \end{array}\right)+{\left(\frac{P_{0}L^{2}}{EI}\right)}^{\;2}\left(\begin{array}{cc} -\frac{1}{700} & \frac{1}{1400}\\ \frac{1}{1400} & -\frac{11}{6300} \end{array}\right)\right]\left(\begin{array}{c} \frac{\Delta Y}{L}\\ \theta_{0} \end{array}\right).$$

(9) $$\delta_{x}=\frac{t_{0}^{2}p_{0}}{12EI}-\frac{1}{2}\left(\begin{array}{cc} \frac{\Delta Y}{L} & \theta_{0} \end{array}\right)\left(\begin{array}{cc} \frac{6}{5} & -\frac{1}{10}\\ -\frac{1}{10} & \frac{2}{15} \end{array}\right)\left(\begin{array}{c} \frac{\Delta Y}{L}\\ \theta_{0} \end{array}\right)-\frac{P_{0}L^{2}}{EI}\left(\begin{array}{cc} \frac{\Delta Y}{L} & \theta_{0} \end{array}\right)\left(\begin{array}{cc} -\frac{1}{700} & \frac{1}{1400}\\ \frac{1}{1400} & -\frac{11}{6300} \end{array}\right)\left(\begin{array}{c} \frac{\Delta Y}{L}\\ \theta_{0} \end{array}\right).$$

As shown in Figure 6, a local coordinate system $O_{n}x_{n}y_{n}$ is established at the tangent direction of the end point of each segment. The local coordinate system of the first segment is set at the fixed end of the bistable beam, and the free-end node of the bistable beam is designated as *N*. In a local coordinate system of one of the three segments, $f_{n}$, $p_{n}$, and $m_{n}$ represent the normalized transverse force, normalized axial force, and normalized bending moment of the node on this segment, respectively. Meanwhile, the corresponding transverse displacement, axial displacement, and end rotation angle are denoted by $\delta_{xn}$, $\delta_{yn}$, and $\alpha_{n}$. The load balance equation for a certain segment of the beam can be listed as follows:

(10) $$\left(\begin{array}{ccc} \cos\theta_{n} & -\sin\theta_{n} & 0\\ \sin\theta_{n} & \cos\theta_{n} & 0\\ \left(1+\delta_{xn}\right) & -\delta_{yn} & 1 \end{array}\right)\left(\begin{array}{c} f_{n}\\ p_{n}\\ m_{n} \end{array}\right)=\left(\begin{array}{c} f_{1}\\ p_{1}\\ m_{i-1} \end{array}\right)$$

The end loads $f_{n}$, $p_{n}$, and $m_{n}$ are normalized to obtain the following:

(11) $$f_{0}=n^{2}f_{1}\begin{array}{ccc} ;\; & & \end{array}P_{0}=n^{2}p_{1}\begin{array}{ccc} ;\; & & \end{array}m_{0}=nm_{n}.$$

Therefore, the geometric constraint equation of the planar compliant beam is as follows:

(12) $$\left\{\begin{array}{c} \left(\begin{array}{c} X_{0}\\ Y_{0} \end{array}\right)=\sum_{i=1}^{n-1}\left[\left(\begin{array}{cc} \cos\theta_{i} & -\sin\theta_{i}\\ \sin\theta_{i} & \cos\theta_{i} \end{array}\right)\left(\begin{array}{c} L_{i}\left(1+\delta_{yi}\right)\\ L_{i}\delta_{yi} \end{array}\right)\right]\\ \theta_{0}=\theta_{n}+\alpha_{n} \end{array}\right.$$

**Figure 7:**
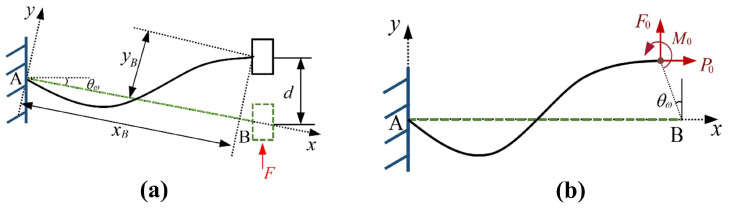
Schematic of mechanical analysis of the bistable beam: (**a**) schematic of the planar beam; (**b**) schematic of the planar beam after coordinate transformation.

After obtaining the geometric constraint equation of the compliant beam, a mechanical analysis of the compliant beam is conducted. As shown in Figure 7, a global coordinate system is established, with the fixed end A of the compliant beam as the coordinate origin. The *x*-axis extends along the length of the beam, and the *y*-axis is perpendicular to the beam. For the guide end B, its transverse displacement is *d*; therefore, the displacement expression for the end of the planar compliant beam can be expressed as follows:

(13) $$\left\{\begin{array}{c} X_{0}=L_{AB}-d\sin\theta_{\omega }\\ Y_{0}=-\;d\cos\theta_{\omega }\\ \theta_{0}=0 \end{array}\right.$$

The transverse force *F* acting on the guide end *N* can be expressed in terms of the end loads ($F_{0}$ and $P_{0}$) of the beam:

(14) $$F=F_{0}\cos\theta_{\omega }+P_{0}\sin\theta_{\omega }$$

Since the bistable beam unit and the bistable rhombic beam unit are structurally concatenated in series, the following governing relationship is satisfied:

(15) $$\left\{\begin{array}{cc} \; & \;\Delta x=\Delta x_{1}+\Delta x_{2}\\ F_{NSM} & =K_{NSM}\Delta x=K_{unit1}\Delta x_{1}=K_{unit2}\Delta x_{2} \end{array}\right.$$

In this context, $\Delta x_{1}$ and $\Delta x_{2}$ represent the displacements of the two negative-stiffness units, while $K_{unit1}$ and $K_{unit2}$ represent the stiffnesses of the bistable beam and bistable rhombic beam, respectively. Additionally, Δx and $K_{NSM}$ represent the displacement and stiffness of the negative-stiffness mechanism. Given this serial connection configuration, the following stiffness equation is derived:

(16) $$K_{NSM}=\frac{K_{unit1}K_{unit2}}{K_{unit1}+K_{unit2}}=\frac{2\left(F_{0}\cos\theta_{\omega }+P_{0}\sin\theta_{\omega }\right)}{\Delta x_{1}+\Delta x_{2}}$$

The positive-stiffness mechanism and the negative-stiffness mechanism are combined through a parallel structure, where the stiffness of the entire resilient mechanism is determined by the principle of stiffness superposition:

(17) $$K=K_{pos}+K_{NSM}=\frac{2Ebt^{3}}{2l_{1}^{3}+l_{2}^{3}}+\frac{2\left(F_{0}\cos\theta_{\omega }+P_{0}\sin\theta_{\omega }\right)}{\Delta x_{1}+\Delta x_{2}}$$

Through theoretical analysis of the positive-stiffness mechanism and negative-stiffness mechanism, and using MATLAB R2023a software to draw schematic diagrams of curves, the curves of the constant-force interval are finally formed.

## 4. Parametric Analysis and Finite-Element Optimization

### 4.1. Parameter Sensitivity Analysis

To clarify the influence law of structural parameters on the performance of the CCFM and determine the initial parameters for optimization simulation, based on the analysis in the previous section, all structural parameters have now been determined (with specific values shown in Table 1). Through parameter sensitivity analysis, the specific influence of each parameter on the constant-force performance is explored, and parameters with significant influence are screened out, laying a foundation for subsequent finite-element optimization.

**Figure 8:**
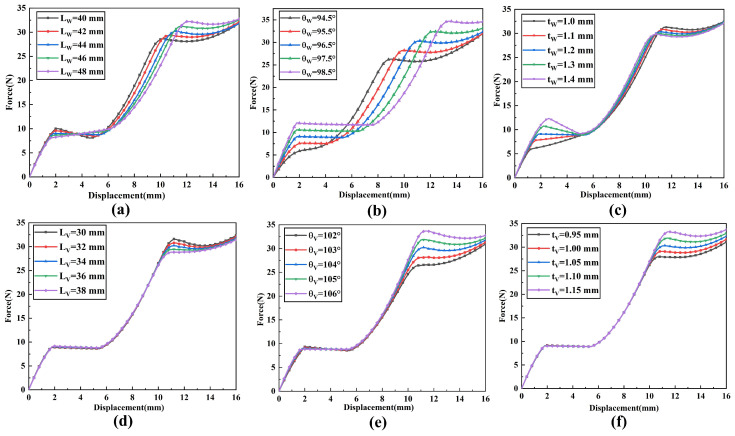
Influence of negative-stiffness mechanism parameters on the constant-force characteristics of the DSCCFM: (**a**) $L_{W}$, (**b**) $\theta_{W}$, (**c**) $t_{W}$, (**d**) $L_{V}$, (**e**) $\theta_{V}$, (**f**) $t_{V}$.

**Figure 9:**
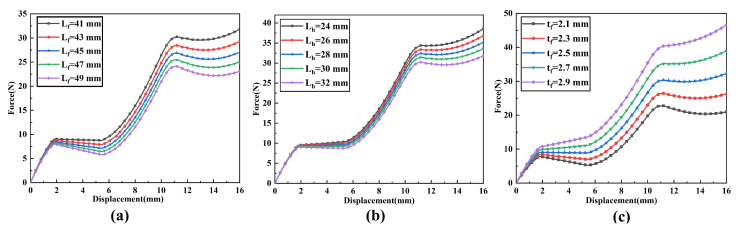
Influence of positive-stiffness mechanism parameters on the constant-force characteristics of the DSCCFM: (**a**) $L_{f}$; (**b**) $\theta_{h}$; (**c**) $t_{f}$.

When analyzing the influence of a single parameter on the constant-force performance, other parameters need to be kept constant. The influences of changes in each structural parameter on the constant-force performance are shown in Figure 8 and Figure 9. Among them, Figure 8 shows that for the bistable beam, as the inclination angle $\theta_{W}$ increases, both the output force and the constant-force range show an increasing trend. The influence of length $L_{W}$ is opposite to that of inclination angle $\theta_{W}$; as the out-of-plane thickness $t_{W}$ increases, the constant-force range narrows, but the output force increases. Moreover, the structural parameters of the bistable beam have an influence on both the first and second stages of the constant force of the constant-force mechanism. The structural parameters of the bistable rhombic beam (as shown in Figure 8) mainly affect the second-stage constant-force performance. An increase in beam length $L_{V}$ leads to a decrease in output force, while an increase in beam thickness $t_{V}$ has the opposite effect. At the same time, an increase in beam inclination angle $\theta_{V}$ significantly enhances both the output force level and the constant-force range of the second-stage constant force. The parameter influences of the positive-stiffness Z-shaped beam are shown in Figure 9; increases in the lengths of the long beam $L_{f}$ and short beam $L_{h}$ both reduce the output force, while beam thickness $t_{f}$ shows the opposite trend. In summary, the results of the parameter sensitivity analysis clearly show that the above nine structural parameters all have key influences on the performance indicators of the constant-force mechanism (output force, constant-force range, and staged performance). Thus, they are identified as the core structural parameters for the subsequent optimization design of the mechanism.

### 4.2. Structural Optimization

**Figure 10:**
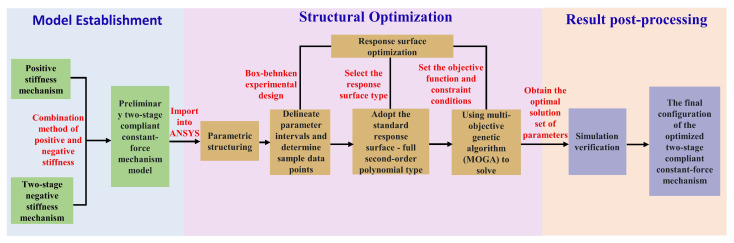
Optimization framework flowchart.

Based on ANSYS 2023 R2 finite-element analysis, a response surface optimization design is conducted for the CCFM. By reasonably setting parameter sample points, screening suitable experimental methods, and selecting appropriate types of response surfaces, the mapping relationship between independent variables and response values is accurately established. Given the significant mutual influence among the mechanism parameters, the MOGA is adopted for global optimization to effectively explore the design space and obtain the optimal solution set. The system optimization framework is shown in Figure 10.

The design goal of a CCFM usually lies in achieving the target constant-force output and maximizing the constant-force range, and the corresponding optimization objectives can be expressed as follows:

(18) $$Q:\left\{\begin{array}{c} F_{a}\left(L_{W},\theta_{W},t_{W},L_{V},\theta_{V},t_{V},L_{f},L_{h},t_{h}\right)=F_{b}\\ \max S_{a}\left(L_{W},\theta_{W},t_{W},L_{V},\theta_{V},t_{V},L_{f},L_{h},t_{h}\right) \end{array}\right.$$

Herein, $F_{a}$ is the output force of the compliant constant-force mechanism, $S_{a}$ is the corresponding constant-force stroke, and it is the desired constant-force output, among which $L_{W}$, $\theta_{W}$, $t_{W}$, $L_{V}$, $\theta_{V}$, $t_{V}$, $L_{f}$, $L_{h}$, and $t_{h}$ are the structural parameters to be optimized.

**Figure 11:**
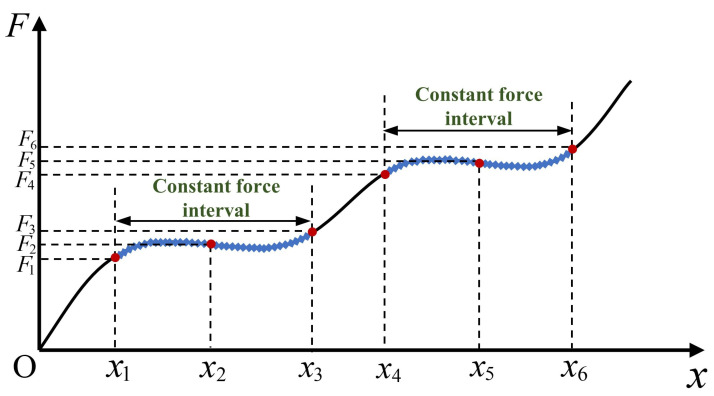
Schematic of sampling points.

Figure 11 shows the force–displacement curve obtained based on finite-element analysis (FEA). A key displacement point $x_{n}$ and its corresponding output force $F_{n}$ are selected. The determination of the constant-force stroke is based on the fitted slope $K_{n}$ of adjacent points; as $K_{n}$ approaches zero, the output force becomes approximately constant within this displacement interval. Meanwhile, the average value of total deformation (TDA) and the maximum value of equivalent stress (ESM) are introduced as auxiliary indicators. A higher TDA value indicates excellent overall compliance and deformation capacity of the mechanism, while a lower ESM value reflects a more uniform stress distribution. Based on this, the rules for establishing the finite-element optimization objective function are as follows:

• Maximization of the constant-force stroke of $\left(x_{3}-x_{1}\right)$ and $\left(x_{6}-x_{4}\right)$;

• Minimization of the gradient of selected points between $x_{1}∼x_{3}$ and $x_{4}∼x_{6}$;

• Maximization of TDA and minimization of ESM for mechanism optimization.

**Figure 12:**
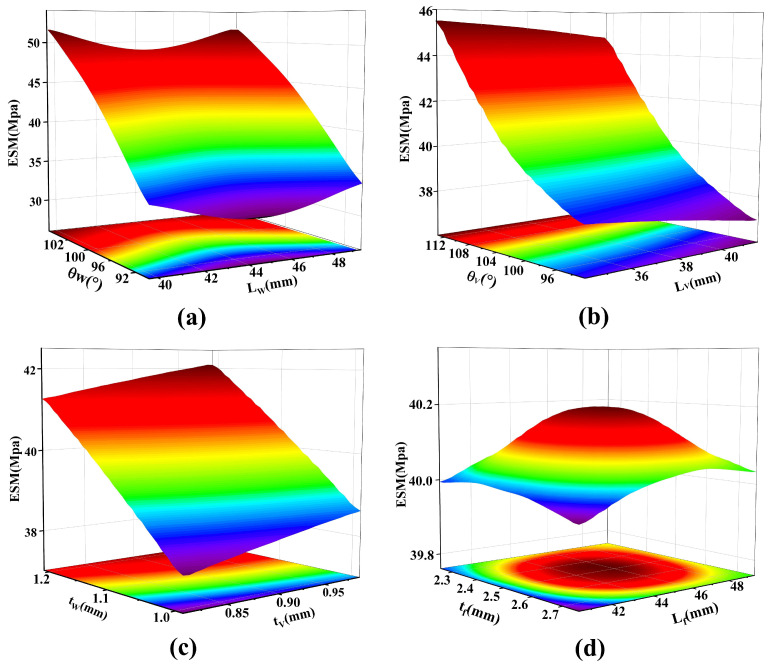
3D response surface of ESM with respect to structural parameters (**a**) $L_{W}$ and $\theta_{W}$, (**b**) $L_{V}$ and $\theta_{V}$, (**c**) $t_{W}$ and $t_{V}$, and (**d**) $L_{f}$ and $t_{f}$.

In the response surface optimization process of the CCFM, first, based on the reasonable variation range of key structural parameters, the Box–Behnken experimental design method is selected for sampling. With its excellent space-filling property, this method can achieve efficient and uniform distribution of sampling points within the set parameter space, effectively avoiding non-convergence of finite-element analysis caused by extreme parameter combinations. Subsequently, a standard second-order polynomial response surface model is selected for fitting, which can fully capture the complex nonlinear relationships and significant interaction effects between design parameters and target performance indicators (such as constant-force range, output force, ESM, etc.). Figure 12 shows the fitted response surface relationship between the ESM and key design parameters, intuitively demonstrating the influence law of parameter changes on ESM.

Based on the analysis results of the 3D response surface, the influence degrees of each optimization parameter on ESM vary. As shown in Figure 12, parameters $L_{W}$, $\theta_{W}$, and $t_{W}$ of the bistable beam have the most significant influence on ESM, followed by parameters $L_{V}$, $\theta_{V}$, and $t_{V}$ of the bistable rhombic beam, while the beam length $L_{f}$ and beam thickness $t_{f}$ of the positive-stiffness mechanism have a relatively smaller influence. To enable the DSCCFM to obtain optimal constant-force characteristics, attention should be paid to the changes in the structural parameters of the bistable beam. Based on the established high-precision 3D response surface model, the objective function is clearly defined, and constraint conditions are set in the optimization module, and the MOGA is adopted for a global optimization solution. The optimization process is carried out through the proper configuration of algorithm parameters. The algorithm then converges to obtain the optimal solution set, from which the final geometric configuration of the optimized structure is determined.

**Figure 13:**
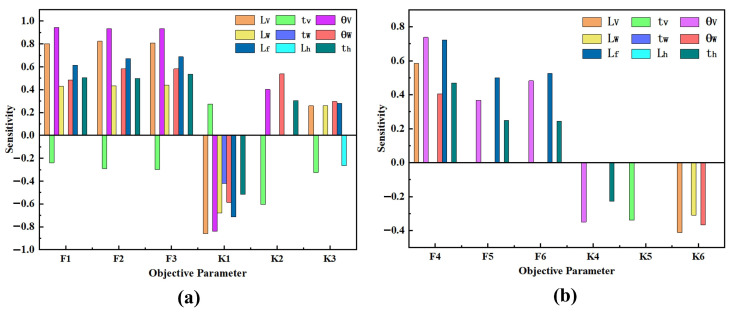
Sensitivity of structural parameters to objective parameters: (**a**) sensitivity in the first-stage constant-force interval; (**b**) sensitivity in the second-stage constant-force interval.

During the output stage of the optimization results, to clarify the differentiated characteristics of the influence of different structural parameters on the objective function, a further parameter sensitivity analysis was conducted, the results of which are shown in Figure 13. Among them, optimization parameters $L_{V}$, $L_{h}$, $\theta_{W}$, and $\theta_{V}$ are more sensitive to the objective function, while parameters $L_{f}$ and $t_{W}$ of the positive-stiffness mechanism have lower sensitivity to the objective function. Therefore, targeted regulation of high-sensitivity parameters can effectively optimize the performance of the constant-force mechanism, simultaneously improving the stability of the output force and expanding the range of effective constant-force stroke.

**Figure 14:**
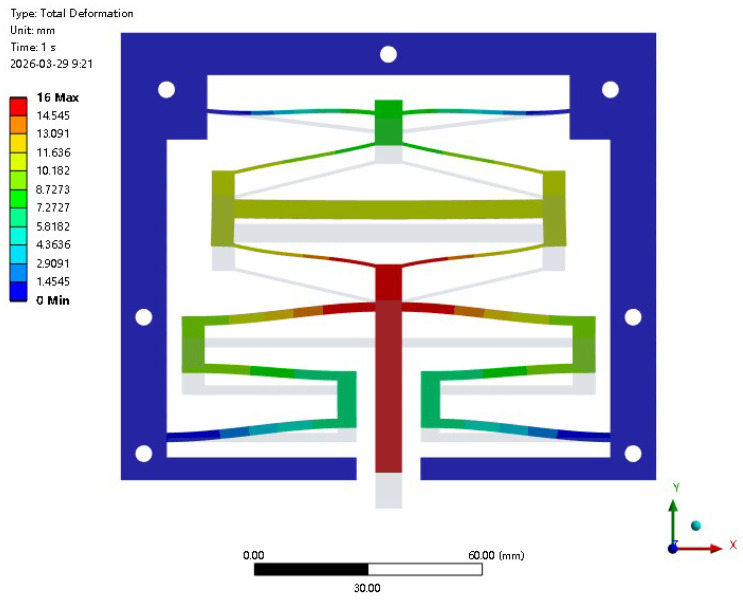
Simulation analysis of the constant-force mechanism.

**Figure 15:**
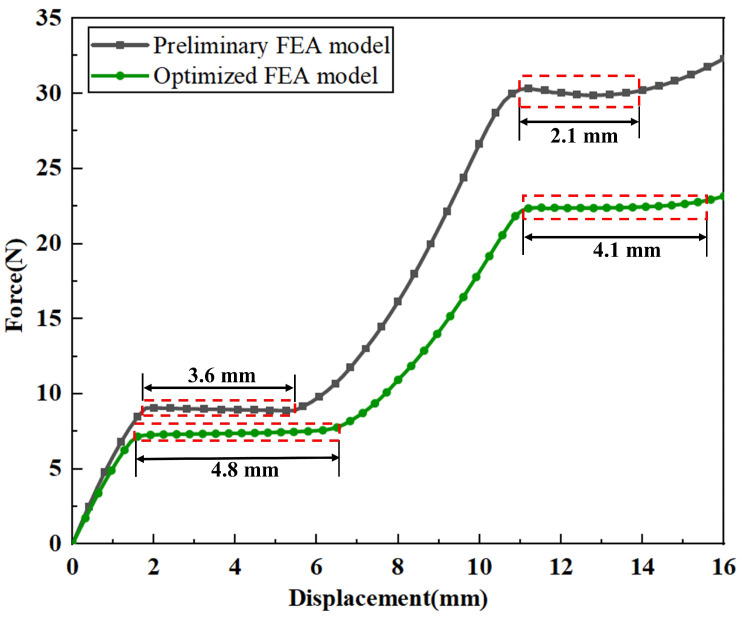
Comparison of constant-force characteristics of the mechanism before and after optimization based on the DSCCFM.

By establishing a multi-objective optimization model, the optimal parameter combination of the CCFM is obtained (see Table 2 for details). Based on the optimized structural parameters, the model of the CCFM was reconstructed and analyzed using finite-element simulation. The material selected for the model was a photosensitive resin. For mesh discretization, an element size of 0.4 mm was adopted, and the model was meshed using SOLID187 tetrahedral elements. The simulation results indicate that the mesh size had a negligible influence on the final convergence, demonstrating that the adopted mesh parameters did not significantly affect the convergence behavior and ensured the stability and reliability of the numerical analysis. In addition, the large deformation behavior of the mechanism was taken into account, while shear deformation effects were neglected, thereby enabling an accurate characterization of its nonlinear mechanical response. In terms of boundary conditions, constraints were applied through the seven fixing holes of the constant-force mechanism, and a displacement load was imposed on the output end of the mechanism in the bottom-to-top direction. The overall deformation contour is shown in Figure 14; a comparison of the force-displacement curves of the constant-force mechanism before and after optimization is shown in Figure 15. The pre-optimization model has dual stages of constant-force output: the first-stage constant-force output value is 9.2 N, with a constant-force interval of 1.8 mm–5.4 mm and a constant-force interval gap of 3.6 mm. The second-stage constant-force output value is 27.2 N, with a constant-force interval of 11.2 mm–13.4 mm and a constant-force interval gap of 2.2 mm. After optimization, the first-stage constant-force output value of the model is 7.3 N, the constant-force interval is expanded to 1.7 mm–6.5 mm, and the constant-force interval gap is increased to 4.8 mm. The second-stage constant-force output value is adjusted to 22.2 N, the constant-force interval is widened to 10.7 mm–14.8 mm, and the constant-force interval gap is increased to 4.1 mm. It can be seen from this that the effective working intervals of the two constant-force stages after optimization have been significantly expanded, the overall constant-force interval gap of the model has increased by 38%, and the constant-force output performance has been remarkably enhanced to a notable extent.

## 5. Experimental Verification

After completing the mechanical design, theoretical modeling, and simulation optimization analysis of the constant-force mechanism, this study further constructed a physical prototype based on the established theoretical model and conducted experimental verification work. The relevant experimental results are described in the remainder of this section.

### 5.1. Experimental Apparatus

The DSCCFM is manufactured using 3D printing and is made of photosensitive resin with a Young’s modulus of 2200 MPa and a Poisson’s ratio of 0.33. The magnitude of the constant force generated by the mechanism depends on the material used; for the designed DSCCFM, photosensitive resin is mainly used in the experiments.

**Figure 16:**
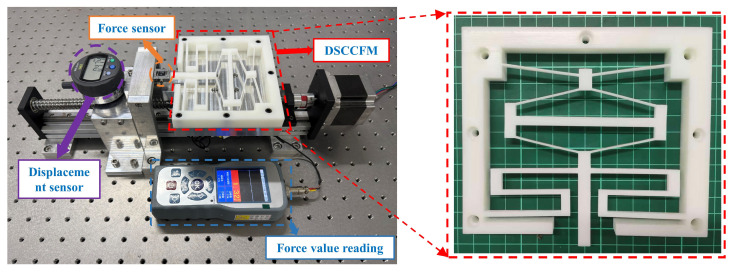
Experimental setup.

The 3D-printed constant-force mechanism was installed on an aerostatic platform for the experiment. The experimental setup is shown in Figure 16, including the constant-force mechanism, micrometer gauge, force sensor, controller, ball screw module, and drive power supply of the stepper motor. The constant-force mechanism was fixed on the slider of the ball screw module via an adapter plate, and the stepper motor was controlled to incrementally and decrementally move along its traveling direction within the range of S = 0–16 mm. The force sensor and the micrometer gauge were used to measure the magnitudes of force and displacement, and the data were recorded.

### 5.2. Experimental Results

**Figure 17:**
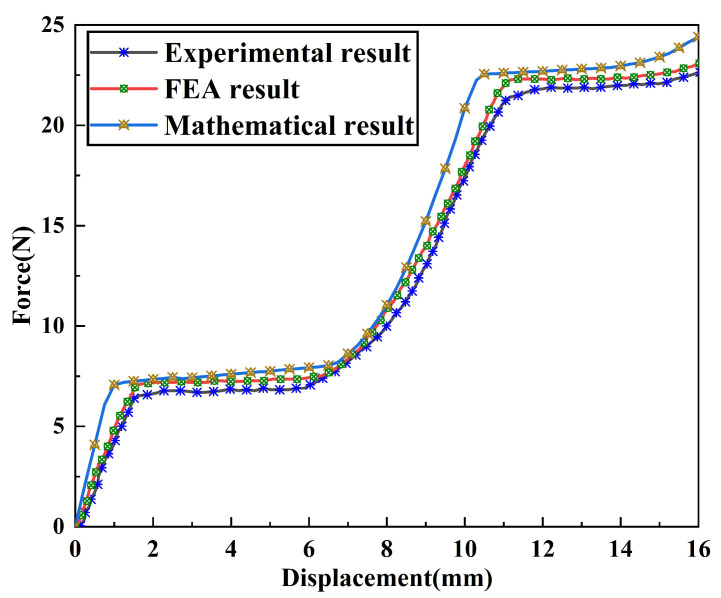
Force–displacement curve of the DSCCFM.

In this section, experimental tests and performance evaluation were performed on the DSCCFM, and comprehensive verification was carried out by combining mathematical modeling, simulation analysis, and experimental data. The force–displacement curve in Figure 17 shows that within the stroke ranges of 2 mm−5.8 mm and 11.6 mm−14.8 mm, the mechanism achieves constant output forces of approximately 6.72 N and 21.91 N, verifying its design function of providing segmented constant force in two stroke intervals. Further comparison shows that the experimental results are in good agreement with the theoretical and simulation results: the error between the experimental values and theoretical values is approximately 8.5%, which is within an acceptable range; the error between the experimental values and simulation values is approximately 4.7%. The deviation between them is mainly attributed to the machining errors of the actual model and the mesh division of the simulation model.

**Figure 18:**
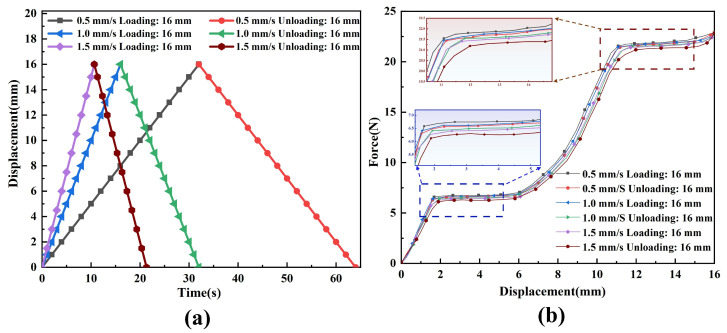
Experimental tests of the constant-force mechanism under different speeds: (**a**) loading–unloading curves of the constant-force mechanism under different speeds; (**b**) force–displacement response curves of the constant-force mechanism under different speeds.

To evaluate the performance stability of the constant-force mechanism under different motion speeds, this study conducted dynamic loading–unloading experiments on the DSCCFM. Within a stroke range of 16 mm, the mechanism was controlled to perform one complete loading–unloading cycle at three different speeds. As shown in Figure 18, during the loading–unloading processes at different speeds, the errors between the measured output force and the expected constant-force value were approximately 3.8%, 4.6%, and 5.8%, respectively. This is because its assembly/alignment errors and measurement errors can also cause discrepancies.

**Figure 19:**
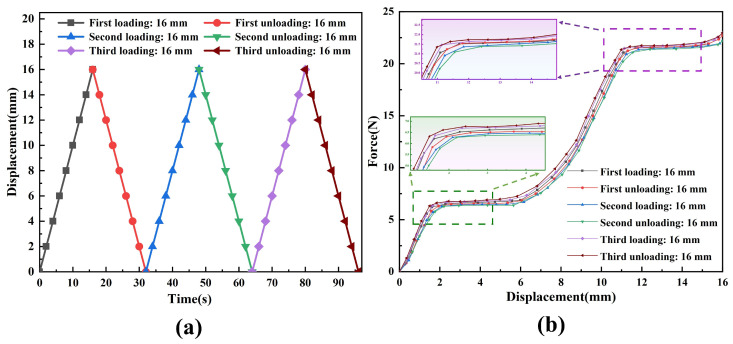
Experimental tests of the constant-force mechanism under cyclic loading–unloading. (**a**) Cyclic loading–unloading curves of the constant-force mechanism. (**b**) Cyclic loading–unloading force–displacement response curves of the constant-force mechanism.

To further investigate the operating performance of the constant-force mechanism during cyclic use at the same speed, experimental tests were conducted on the constant-force mechanism under three repeated loading–unloading modes. The experimental results, showcased in Figure 19, reveal that during the three loading–unloading processes, the relative errors between the measured output force and the expected output force are 3.9%, 4.5%, and 4.8%, respectively. The above errors mainly come from the uncertainties introduced during the experimental measurement process and assembly.

Based on the force–displacement response curves obtained from the aforementioned cyclic loading–unloading experiments (as shown in Figure 19), a comparative analysis of the experimental data reveals that these curves clearly reveal a non-coincidence phenomenon between the loading and unloading paths, namely hysteresis. This phenomenon is mainly manifested as an obvious lag of the unloading curve relative to the loading curve. From the perspective of the causes of the lag, it is primarily attributed to the intrinsic dissipation behavior of the material under cyclic stress, such as elastic hysteresis and microplastic deformation. To quantitatively evaluate the hysteresis performance of the mechanism, this study proposes using the force-hysteresis-clearance-to-stroke ratio as the evaluation index. Calculations based on the experimental data show that this ratio is 0.0255, 0.0228, and 0.0278 during the three cycles. This experimental result clearly confirms that the proposed DSCCFM has a low hysteresis error characteristic, which effectively improves the positioning accuracy and dynamic control reliability during the operation process, making it more suitable for applications such as micro/nano-scale precision operation, cell clamping, and flexible end effectors with small motion consistency and error.

## 6. Conclusions

This study proposes a CCFM capable of producing a dual-stage constant-force output based on the combination of positive and negative stiffness. The mechanism integrates bistable beams, bistable rhombic beams, and Z-shaped beams, which provide negative and positive stiffness, respectively. Theoretical modeling combines the pseudo-rigid-body model for the positive-stiffness component and the chained beam constraint model for the negative-stiffness unit. To enhance the constant-force stroke, the mechanism is optimized using ANSYS response surface methodology and a multi-objective genetic algorithm, followed by structural simulation and experimental validation. The results show good agreement among the theoretical, simulation, and experimental data. The DSCCFM delivers constant output forces of 6.72 N and 21.91 N over stroke ranges of 2–5.8 mm and 11.6–14.8 mm, respectively. Compared with single-stage CCFMs, the proposed design offers a dual-stage output with improved versatility. Moreover, it achieves larger effective strokes than existing dual-stage mechanisms—3.8 mm in the first stage and 3.2 mm in the second—demonstrating enhanced large-stroke capability. This makes the DSCCFM well-suited for applications such as multi-stage constant-force operations, cell clamping, and micro/nano-scale manipulation.

## Figures and Tables

**Table 1:** Initial parameters of the constant-force mechanism.

Parameter	$L_{W}$	$\theta_{W}$	$t_{W}$	$L_{V}$	$\theta_{V}$	$t_{V}$	$L_{f}$	$L_{h}$	$t_{h}$
Value	44 mm	96.5°	1.2 mm	34 mm	103.5°	1.0 mm	41 mm	34 mm	2.55 mm

**Table 2:** Optimized parameters of the constant-force mechanism.

Parameter	$L_{W}$	$\theta_{W}$	$t_{W}$	$L_{V}$	$\theta_{V}$	$t_{V}$	$L_{f}$	$L_{h}$	$t_{h}$
Value	44.5 mm	97°	1.1 mm	38 mm	103°	0.9 mm	45 mm	35 mm	2.5 mm

## Data Availability

The data that support the findings of this study are available from the corresponding author upon reasonable request.

## Abbreviations

The following abbreviations are used in this manuscript:

CCFM	Compliant constant-force mechanism
DSCCFM	Dual-stage compliant constant-force mechanism
CBCM	Chained-beam constraint model
PRBM	Pseudo-rigid-body model
MOGA	Multi-objective genetic algorith
ATD	Average value of total deformation
ESM	Maximum value of equivalent stress
